# [^123^]FP-CIT SPECT scans initially rated as normal became abnormal over time in patients with probable dementia with Lewy bodies

**DOI:** 10.1007/s00259-016-3312-x

**Published:** 2016-01-30

**Authors:** J. J. van der Zande, J. Booij, P. Scheltens, P. G. H. M. Raijmakers, A. W. Lemstra

**Affiliations:** VU Medical Center Alzheimer Center, De Boelelaan 1118, 1081 HZ Amsterdam, The Netherlands; Department of Nuclear Medicine, Academic Medical Center, Amsterdam, The Netherlands; Department of Nuclear Medicine, VU Medical Center, Amsterdam, The Netherlands

**Keywords:** Dementia with Lewy bodies, Neuroimaging, Dopamine transporter, [^123^I]FP-CIT SPECT

## Abstract

**Purpose:**

Decreased striatal dopamine transporter (DAT) binding on SPECT imaging is a strong biomarker for the diagnosis of dementia with Lewy bodies (DLB). There is still a lot of uncertainty about patients meeting the clinical criteria for probable DLB who have a normal DAT SPECT scan (DLB/S−). The aim of this study was to describe the clinical and imaging follow-up in these patients, and compare them to DLB patients with abnormal baseline scans (DLB/S+).

**Methods:**

DLB patients who underwent DAT imaging ([^123^I]FP-CIT SPECT) were selected from the Amsterdam Dementia Cohort. All [^123^I]FP-CIT SPECT scans were evaluated independently by two nuclear medicine physicians and in patients with normal scans follow-up imaging was obtained. We matched DLB/S-− patients for age and disease duration to DLB/S+ patients and compared their clinical characteristics.

**Results:**

Of 67 [^123^I]FP-CIT SPECT scans, 7 (10.4 %) were rated as normal. In five DLB/S− patients, a second [^123^I]FP-CIT SPECT was performed (after on average 1.5 years) and these scans were all abnormal. No significant differences in clinical characteristics were found at baseline. DLB/S− patients could be expected to have a better MMSE score after 1 year.

**Conclusion:**

This study was the first to investigate DLB patients with the initial [^123^I]FP-CIT SPECT scan rated as normal and subsequent scans during disease progression rated as abnormal. We hypothesize that DLB/S− scans could represent a relatively rare DLB subtype with possibly a different severity or spread of alpha-synuclein pathology (“neocortical predominant subtype”). In clinical practice, if an alternative diagnosis is not imminent in a DLB/S− patient, repeating [^123^I]FP-CIT SPECT should be considered.

## Introduction

Dementia with Lewy bodies (DLB) is the second most common neurodegenerative dementia in ageing populations [1]. The pathological hallmark of DLB is the presence of aggregations of alpha-synuclein in Lewy Bodies and Lewy neurites in the brain. The core clinical features of DLB consist of progressive cognitive decline in combination with extrapyramidal signs, hallucinations and/or fluctuations of cognition [2]. DLB is a heterogeneous disease with a range of symptoms that can present in various ways in individual patients. DLB has considerable overlap with Alzheimer’s disease (AD) and Parkinson’s disease (PD), both clinically and pathologically. Therefore, diagnosing DLB can be challenging.

In 2005, imaging of the dopamine transporter (DAT) with SPECT was added to the diagnostic criteria for DLB as a supportive feature [3]. ^123^I-Labelled *N*-ω-fluoropropyl-2β-carbomethoxy-3β-(4-iodophenyl)nortropane ([^123^I]FP-CIT) SPECT measures the integrity of dopaminergic terminals and is a well-validated tool for detecting degeneration of nigrostriatal dopaminergic cells in PD and DLB [4–6].

A meta-analysis of the diagnostic value of [^123^I]FP-CIT SPECT showed a sensitivity of 86.5 % and a specificity of 93 % for detecting DLB [7]. Most of the included studies compared the results of [^123^I]FP-CIT SPECT imaging with the clinical diagnosis, which was used as the gold standard. A study comparing [^123^I]FP-CIT SPECT and autopsy findings found a sensitivity of 86 % and a specificity of 83 %, and of 100 and 92 % if analysed semiquantitatively [8]. A recently published Cochrane review indicates that a normal [^123^I]FP-CIT SPECT scan may exclude the diagnosis in patients with dementia and a high clinical suspicion of DLB [9].

However, occasionally in clinical practice a patient fulfilling the criteria for probable DLB has an [^123^I]FP-CIT SPECT scan rated as “normal” or “negative”. There has been very little research concerning such patients. These may be patients with true DLB patients with negative scans or patients with an aberrant clinical diagnosis suffering from another type of dementia. A previous study has shown that in some patients with normal ante-mortem [^123^I]FP-CIT SPECT scans, DLB is found on autopsy [10]. It has been suggested that this could be due to a variation in the distribution of Lewy body pathology, with preferential involvement of cortical brain areas [11].

In this study, we set out to investigate patients with probable DLB and negative [^123^I]FP-CIT SPECT (DLB/S−) scans in the Amsterdam Dementia Cohort. We explored their clinical characteristics compared with those in DLB patients with abnormal (DLB/S+) baseline scans. We describe the clinical follow-up and the results of repeated [^123^I]FP-CIT SPECT imaging.

## Materials and methods

### Patient selection and study design

Patients were selected from the Amsterdam Dementia Cohort, a prospective clinical dementia cohort of patients who visited the memory clinic of the VU University Medical Center for dementia screening between 2004 and July 2014 [12]. All patients underwent extensive standardized dementia screening, including a physical and neurological examination, neuropsychological test examinations, neuropsychiatric inventory (NPI), EEG, MRI, routine laboratory blood tests, and a lumbar puncture. Diagnoses were made by consensus in a multidisciplinary team according to the current international diagnostic criteria for various dementia syndromes. For DLB, the international consensus criteria of McKeith et al. were used [3]. Additional [^123^I]FP-CIT SPECT scans were performed at the discretion of the clinical team.

For this case-control study, patients with a clinical diagnosis of probable DLB in whom [^123^I]FP-CIT SPECT imaging was performed, were selected. Stable diagnoses of probable DLB had to be confirmed during follow-up. Follow-up [^123^I]FP-CIT SPECT scans were obtained. For clinical comparison, the DLB/S− patients were matched for age and disease duration at a ratio of 1:2 in relation to DLB/S+ patients.

### Clinical outcome measures

The following parameters were retrieved from our institution’s prospectively collected database: presence of visual hallucinations as reflected by a positive score on the NPI subitem hallucinations [13], presence of extrapyramidal signs based on a preformatted checklist, and global cognition assessed by the Mini Mental State examination (MMSE). Data on medication use and care-giver information about the presence of fluctuations in cognition and symptoms of rapid eye movement (REM) sleep behaviour disorder (RBD) were derived retrospectively from patient charts. The procedure for EEG evaluation is described elsewhere [12]; we dichotomized the patients according to the normality or abnormality of the results. Regarding MRI, hippocampal atrophy (MTA score [14]), global atrophy and white matter hyperintensities according to the Fazekas scale were compared. If cerebrospinal fluid (CSF) biomarker analysis was available, tau/Aβ42 ratios were calculated, and a ratio of >0.52 was considered to represent an AD profile [15]. Patients were followed with annual assessments for as long as this was feasible for clinical and/or research purposes. We obtained MMSE scores at follow-up visits as a global measure of cognitive decline.

### Imaging

The SPECT imaging protocol has been described in detail previously [16]. Imaging was performed according to the guidelines of the European Association of Nuclear Medicine [17] using the validated DAT radiotracer [^123^I]FP-CIT. [^123^I]FP-CIT was injected intravenously 3 h before imaging at an approximate dose of 185 MBq (specific activity >185 MBq/nmol; radiochemical purity >99 %). Patients were scanned using a dual-head gamma camera (model E.Cam; Siemens, Munich, Germany) with a fan-beam collimator. Images were reconstructed using filtered back projection with a Butterworth filter (order 8, cut-off 0.6 cycles/cm).

Individual SPECT images were analysed using a standard template with five regions of interest of fixed size for the left and right head of the caudate nucleus, left and right putamen and occipital cortex, as described previously [16]. Specific to nonspecific DAT binding ratios (BRs) were calculated for the left and right putamen and head of the bilateral caudate nuclei, using the occipital cortex as the reference area. For evaluation of reproducibility of scan readings, 42 [^123^I]FP-CIT SPECT scans of DLB and non-DLB dementia patients (mainly AD or frontotemporal dementia) without any clinical information except the age of the patient were evaluated by a second independent nuclear medicine physician. In doubtful cases, the final assessment was made at a consensus meeting with both physicians taking into account visual assessments and age-matched BRs [16].

### Analysis

Data were analysed with IBM SPSS statistics for Windows, version 20 (IBM Corp, Armonk, NY). Descriptive statistics were calculated for group comparisons. All continuous variables are reported as medians (range), categorical variables are presented as their actual values and as percentages of the group totals. For nonnormally distributed data, nonparametric tests were used; Fisher’s exact test for categorical variables and the Mann-Whitney *U* test for continuous variables. A *p* value less than 0.05 was considered statistically significant. For reproducibility analyses, interobserver variation was assessed using Cohen’s kappa in 42 [^123^I]FP-CIT SPECT scans [18].

### Ethics approval

Written informed consent for use of their clinical data for research purposes was obtained from all patients. The medical ethics committee of the VU University Medical Center approved the study.

## Results

From 2008 until 2014, 67 of 175 patients with a diagnosis of probable DLB underwent [^123^I]FP-CIT SPECT imaging. After the first visual assessment (Cohen’s kappa 0.7) and consensus meeting, the [^123^I]FP-CIT SPECT scan was normal in 7 (10.4 %) of the 67 patients with probable DLB. One of these DLB/S− patient was excluded from further analysis because of a follow-up duration shorter than 6 months, leaving six patients in the DLB/S− group.

**Fig. 1:**
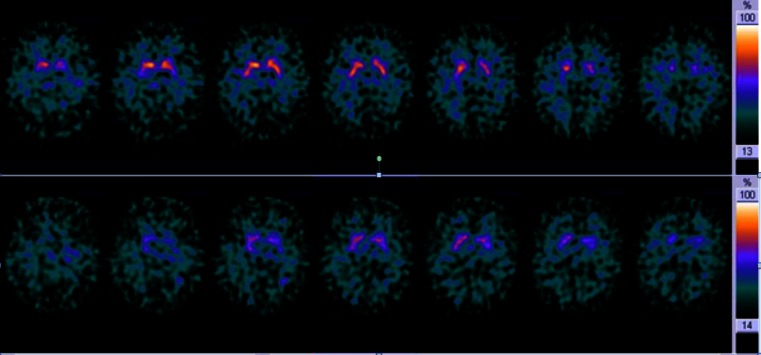
*Upper panel*: [^123^I]FP-CIT SPECT scan (transverse slices) acquired in 2011 (binding ratios: right caudate nucleus 2.62, left caudate nucleus 2.56, right putamen 2.37, and left putamen 2.21). *Lower panel*: follow-up [^123^I]FP-CIT SPECT scan acquired in 2012 shows lower tracer binding (binding ratios: 1.82, 1.84, 1.95, and 1.70, respectively)

**Fig. 2:**
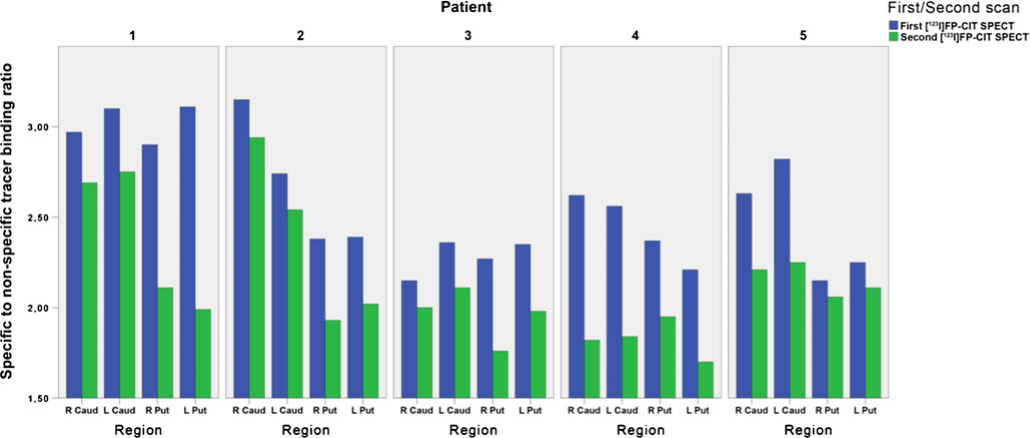
Tracer binding ratios in four regions of interest in five DLB patients with an initially normal [^123^I]FP-CIT SPECT scan (*blue*). The binding ratios are lower on the second [^123^I]FP-CIT SPECT scan (*green*)

Clinical diagnosis of DLB was reconfirmed during follow-up (median 24 months, interquartile range 8 – 36 months) in all patients. Five of the six DLB/S− patients underwent a second [^123^I]FP-CIT SPECT scan during the follow-up period. These scans were performed because of diagnostic uncertainty, e.g. clinically probable DLB but a normal initial [^123^I]FP-CIT SPECT scan. The remaining patient was burdened by having to travel to Amsterdam and was referred back to the neurologist in his home town after 18 months without repeated SPECT imaging. However, clinical criteria remained and there were no signs pointing to an alternative diagnosis. The five follow-up scans were all reported as abnormal, consistent with a diagnosis of DLB. The median time between the first and second scan was 18 months (range 9 – 38 months). [^123^I]FP-CIT SPECT imaging in an example patient with an initially normal scan and an abnormal follow-up scan is shown in Fig. 1. Changes in striatal BRs between the first and second [^123^I]FP-CIT SPECT scan in five DLB/S− patients are summarized in Fig. 2.

The clinical characteristics at baseline in 6 DLB/S− patients and 12 DLB/S+ patients are summarized in Table 1. There were no significant differences in education, sex or medication use. In the DLB/S− group, two patients were taking antidepressants and one was taking a cholinesterase inhibitor. In the DLB/S+ group, one patient was taking an antidepressant, three were taking cholinesterase inhibitors, and one was taking levodopa. No differences in MMSE were found at baseline. DLB/S− patients less often showed extrapyramidal signs, although the difference was not statistically significant in this group (*p*=0.11). Two of the DLB/S− patients did present with signs of parkinsonism: one patient with rigidity, tremor and slow gait, and the other with diminished arm swing and tremor. The presence of hallucinations, RBD and fluctuations of cognition did not differ between the groups. There were no significant differences in EEG, MRI and CSF findings between the two groups. None of the patients in whom a lumbar puncture was performed (including the patient without a follow-up [^123^I]FP-CIT SPECT scan) had a tau/Aβ42 ratio >0.52, indicating that there was no important (concomitant) AD pathology in these patients.

**Table 1:** Demographics and clinical characteristics

	DLB/S−^a* ^	DLB/S+^b* ^
Male gender, *n* (%)	6 (100)	8 (67)
Age (years), median (range)	72 (57 – 83)	73 (56 – 80)
Disease duration (years), median (range)	3.5 (1 – 8)	3 (0 – 12)
MMSE score, median (range)	22 (16 – 27)	19 (10 – 29)
Hallucinations, *n* (%)	5 (83)	6 (50)
Cognitive fluctuations, *n* (%)	3 (50)	7 (87) (*n* = 8)
Signs of RBD, *n* (%)	6 (100)	6 (60) (*n* = 10)
Extrapyramidal signs, *n* (%)	2 (33)	10 (83)
Tremor	2 (33)	4 (33)
Rigidity	1 (17)	7 (58)
Bradykinesia	2 (33)	6 (50)
EEG abnormal, *n* (%)	5 (83)	9 (90) (*n* = 10)
MRI, *n* (%)		
Temporal atrophy (MTA ≥2)	1 (17)	3 (25)
Global atrophy ≥2	0	2 (17)
Fazekas score ≥2	1 (17)	2 (18)
CSF tau/Aβ42 ratio, median (range)	0.32 (0.24 – 0.44) (*n* = 5)	0.39 (0.19 – 0.49) (*n* = 4)
Follow-up MMSE score at 1 year, median (range)	27 (23 – 28) (*n* = 5)	22 (17 – 25) (*n* = 5)

MMSE Mini Mental State examination, *RBD* REM sleep behaviour disorder, *CSF* cerebrospinal fluid, *MTA* medial temporal lobe atrophy score, *MRI* magnetic resonance imaging

^a^Patients with dementia with Lewy Bodies and a normal baseline [^123^I]FP-CIT SPECT scan; (*n* = 6, unless otherwise specified)

^b^Patients with dementia with Lewy bodies and an abnormal baseline [^123^I]FP-CIT SPECT; (*n* = 12, unless otherwise specified)

* no statistically significant differences were found, *p*-values>0.05

After 1 year MMSE scores were available in 83 % of the DLB/S− patients and 42 % of DLB/S+ patients. Although patients were regularly followed in the outpatient department, neuropsychological testing was not performed routinely, as in some patients this caused too much psychological stress. Therefore, the statistical significance of differences could not be reliably calculated for MMSE scores. There was a trend for a higher MMSE score in the DLB/S− patients.

## Discussion

To our knowledge, this is the first study evaluating the follow-up in DLB patients with a negative [^123^I]FP-CIT SPECT scan with repeated imaging available. Importantly, all follow-up [^123^I]FP-CIT SPECT scans (performed on average 1.5 years after the baseline scan) were scored as abnormal, consistent with the clinical diagnosis of DLB. This finding confirms that striatal DAT imaging can be normal in some DLB patients, who may develop a significant nigrostriatal dopaminergic deficit later in the course of their disease.

We found normal [^123^I]FP-CIT SPECT scans in 10 % of our cohort of patients with probable DLB. This is in accordance with previous reports [5, 10, 11, 19]. There were no significant differences between DLB/S− and DLB/S+ patients in clinical features of DLB such as dementia severity, extrapyramidal signs, hallucinations, fluctuations or RBD. The presence of extrapyramidal signs in two DLB/S− patients is remarkable, since a recent study has demonstrated a correlation between abnormal [^123^I]FP-CIT SPECT scans and extrapyramidal signs in DLB [20]. Neither of these two patients was taking medication that could have caused parkinsonism or had extensive vascular abnormalities (Fazekas scores <2). Based on follow-up MMSE score, the rate of cognitive decline seemed slower in DLB/S− patients. These findings should of course be interpreted with caution since numbers were small and there was loss to follow-up, particularly in the DLB/S+ group.

Normal [^123^I]FP-CIT SPECT scans have been found in a small series of patients with (autopsy-proven) DLB, but the explanation for this finding is not yet entirely clear [10, 11, 19, 20]. In PD, patients with a clinical diagnosis and negative DAT SPECT imaging have been more extensively studied and known as SWEDD (scan without evidence of dopaminergic deficit) subjects. However, a recent study of SWEDD subjects showed minimal changes in striatal BRs on repeated imaging during follow-up. The authors report that such patients are unlikely to suffer from idiopathic PD [21]. In this respect, our DLB/S− patients differed from the SWEDD PD patients, since in our group [^123^I]FP-CIT SPECT scans did become abnormal over time and the clinical diagnosis was and remained DLB.

The most likely explanation for the normal striatal DAT scans in our DLB patients is that DLB/S− patients do have DLB, but with relative sparing of the dopaminergic neurons in the substantia nigra at the time of the scan. In the post-mortem study mentioned above, two patients with autopsy-proven DLB with a visually normal [^123^I]FP-CIT SPECT scan exhibited a higher density of nigral neurons than patients with an abnormal [^123^I]FP-CIT SPECT scan [10]. In these two patients, the time between imaging and death was 3.5 years. Although the [^123^I]FP-CIT striatal BRs were in the same range as in AD patients, the neuronal density in the substantia nigra (assessed post mortem) was on average somewhat lower than in AD patients. Therefore, a possible explanation for the sparing of nigral neurons could be in the pattern of disease progression. Braak et al. described the caudal-to-rostral progression pattern of alpha-synucleinopathy in PD patients, with pathology starting in the dorsolateral medulla oblongata and expanding to the neocortex [22]. In DLB, three pathological subtypes have been described based on the distribution of Lewy bodies: the brainstem, limbic and neocortical predominant subtypes. It has been reported that not all DLB patients fit the Braak staging system [23, 24]. The DLB/S− patients could represent a neocortical subtype of DLB with progression rostral-to-caudal as suggested by Siepel et al. [11], who described three DLB patients during clinical follow-up with a negative [^123^I]FP-CIT SPECT scan. Our data could support this disease progression pattern by the initially negative, but abnormal follow-up striatal DAT imaging. The progressive loss of nigrostriatal dopaminergic projections in our DLB patients with an initial normal [^123^I]FP-CIT SPECT scan is clearly illustrated in Fig. 2 that shows the decline in striatal tracer binding in all patients undergoing a second [^123^I]FP-CIT SPECT scan.

A strength of this study is that all [^123^I]FP-CIT SPECT scans were independently assessed by two nuclear medicine physicians, who had great experience in the field of DAT SPECT imaging in PD and DLB [6, 16]. This, and the fact that the striatal BRs were taken into account to rate the images, minimized the chance that the DLB/S− scans were erroneously interpreted as normal. The patient group was clinically well characterized and had sufficient follow-up to guarantee diagnostic certainty. In almost all (five of six) DLB/S− patients, CSF was analysed for AD biomarkers, and in none of these patients was the pathology consistent with AD.

The study was limited by the small group size, that was a result of the very low prevalence of normal DAT SPECT scans in DLB patients, and by the fact that some of the clinical data were obtained retrospectively. [^123^I]FP-CIT SPECT scans were only performed in selected patients with probable DLB from our memory clinic, which could have influenced the percentage of normal scans. One DLB/S− patient did not receive follow-up imaging and could still have had a normal scan on follow-up. This patient did have sufficient clinical follow-up meeting the criteria for probable DLB and was therefore not excluded from the clinical comparison. Parkinsonism and RBD were not rated with standardized scales or confirmed with polysomnography. However, the majority of clinical data were collected prospectively in a standardized manner, and assessed by a group of clinical experts. Although a few patients were receiving medication that may have influenced the binding of [^123^I]FP-CIT (mainly anti-depressants and cholinesterase inhibitors), these changes have been reported to be very small and unlikely to influence the overall assessment of the scans [25, 26]. Finally, this study was limited by the fact that [^123^I]FP-CIT SPECT images were evaluated in relation to the clinical diagnosis and not to the tissue-confirmed diagnosis.

Recommendations for further research would be to more extensively study the clinical features of DLB/S− patients. Differences with regard to response to symptomatic treatment, disease course and survival in this group of patients should be further elucidated. Extended clinicopathological studies are needed to relate [^123^I]FP-CIT SPECT negativity to severity and spread of alpha-synuclein pathology. In clinical practice, in our opinion, a normal [^123^I]FP-CIT SPECT scan should not be a reason to reject a diagnosis of DLB in a patient fulfilling the criteria for probable DLB. In line with our present findings, repeating [^123^I]FP-CIT SPECT further in the course of the disease should be considered to support the diagnosis of DLB. Furthermore, cardiac scintigraphy with [^123^I]MIBG to visualize cardiac sympathetic dysfunction has shown good diagnostic ability for the detection of DLB [27, 28], even in early stages of the disease. Its use has not been widely implemented, partly due to practical issues. Since the differential diagnosis of DLB extends beyond AD also including diseases such as corticobasal degeneration, progressive supranuclear palsy and frontotemporal dementia, disorders in which [^123^I]FP-CIT SPECT scans can also be positive, it could be of interest to investigate the clinical value of [^123^I]MIBG cardiac scintigraphy in DLB/S− patients.

### Conclusion

This study was first to investigate a subset of DLB patients with the initial [^123^I]FP-CIT SPECT scan rated as normal and subsequent scans during disease progression rated as abnormal. Consequently, a normal [^123^I]FP-CIT SPECT in a patient with probable DLB should not be a reason to reject the clinical diagnosis of DLB immediately. If an alternative diagnosis is not imminent, repeated [^123^I]FP-CIT SPECT imaging should be considered. We hypothesize that a negative [^123^I]FP-CIT SPECT scan may represent a subtype of DLB with a different severity or spread of alpha-synuclein pathology. Further research is needed to investigate this hypothesis.
